# Genome-Wide Association Study of Polymorphisms Predisposing to Bronchiolitis

**DOI:** 10.1038/srep41653

**Published:** 2017-01-31

**Authors:** Anu Pasanen, Minna K. Karjalainen, Louis Bont, Eija Piippo-Savolainen, Marja Ruotsalainen, Emma Goksör, Kuldeep Kumawat, Hennie Hodemaekers, Kirsi Nuolivirta, Tuomas Jartti, Göran Wennergren, Mikko Hallman, Mika Rämet, Matti Korppi

**Affiliations:** 1 PEDEGO Research Unit, Medical Research Center Oulu, University of Oulu, and Department of Children and Adolescents, Oulu University Hospital, Oulu, Finland; 2 Wilhelmina Children’s Hospital, University Medical Center Utrecht, Utrecht, The Netherlands; 3 Kuopio University Hospital, Pediatrics, University of Eastern Finland, Kuopio, Finland; 4 Department of Pediatrics, University of Gothenburg, Queen Silvia Children’s Hospital, Gothenburg, Sweden; 5 Department of Immunology, Laboratory of Translational Immunology, University Medical Center Utrecht, Utrecht, The Netherlands; 6 RIVM, National Institute for Public Health and the Environment, GZB, Center for Health Protection, Bilthoven, The Netherlands; 7 Department of Pediatrics, Seinäjoki Central Hospital, Seinäjoki, Finland; 8 Department of Pediatrics, University of Turku and Turku University Hospital, Turku, Finland; 9 BioMediTech, University of Tampere, Tampere, Finland; 10 Center for Child Health Research, Tampere University and Tampere University Hospital, Tampere, Finland

## Abstract

Bronchiolitis is a major cause of hospitalization among infants. Severe bronchiolitis is associated with later asthma, suggesting a common genetic predisposition. Genetic background of bronchiolitis is not well characterized. To identify polymorphisms associated with bronchiolitis, we conducted a genome-wide association study (GWAS) in which 5,300,000 single nucleotide polymorphisms (SNPs) were tested for association in a Finnish–Swedish population of 217 children hospitalized for bronchiolitis and 778 controls. The most promising SNPs (n = 77) were genotyped in a Dutch replication population of 416 cases and 432 controls. Finally, we used a set of 202 Finnish bronchiolitis cases to further investigate candidate SNPs. We did not detect genome-wide significant associations, but several suggestive association signals (*p* < 10^−5^) were observed in the GWAS. In the replication population, three SNPs were nominally associated (*p* < 0.05). Of them, rs269094 was an expression quantitative trait locus (eQTL) for *KCND3*, previously shown to be associated with occupational asthma. In the additional set of Finnish cases, the association for another SNP (rs9591920) within a noncoding RNA locus was further strengthened. Our results provide a first genome-wide examination of the genetics underlying bronchiolitis. These preliminary findings require further validation in a larger sample size.

Bronchiolitis is a common lower respiratory infection (LRI) that primarily affects children under two years of age^1^,^2^. Respiratory syncytial virus (RSV) is recognized as the most important causative agent of bronchiolitis worldwide^3^. Typically, RSV epidemics occur in annual cycles and peak during the cold season in temperate climates and during the rainy season in warm climates^4^.

During RSV epidemics, most infants become infected and develop mild disease. However, a fraction of children, between 2% and 3% of an age cohort, develop severe bronchiolitis that requires hospitalization^5^. RSV bronchiolitis is the most common infection-related cause of hospitalization among infants and young children in developed countries^6^. The only means of prevention is injecting high-risk children monthly with anti-RSV monoclonal antibody^7^. There is no vaccine or effective antiviral therapy available for RSV infection. Thus, treatment of bronchiolitis is mainly supportive and includes supplying oxygen and maintaining hydration^8^.

There are several congenital and environmental risk factors, such as prematurity, immune deficiency, congenital heart disease, and tobacco smoke exposure, that predispose children to severe bronchiolitis^4^. However, the risk factors do not completely explain the variation in disease severity, and host genetic factors likely contribute to the severity of viral bronchiolitis. Some evidence of genetic susceptibility was provided by a twin study that showed a higher concordance in hospitalization rates between identical twins than in fraternal twins^9^. That study estimated the heritability of bronchiolitis to be 22%.

Genetic studies of susceptibility to bronchiolitis have mostly focused on variants in selected candidate genes, often related to immunity. A few studies have evaluated the relevance of larger groups of genes and have linked variations in the severity of RSV disease with innate immunity–related genes and the *IL13/IL4* locus in the 5q31 cytokine cluster^10^,^11^. Moreover, genetic studies have demonstrated RSV bronchiolitis–associated loci in genes encoding proteins such as toll-like receptors (TLRs), surfactant protein D (SFTPD), Vitamin D receptor (VDR), and various cytokines, of which some have been suggested in more than one study^3^,^10^,^12^,^13^. However, most genetic associations have not been replicated in subsequent studies.

Severe viral bronchiolitis during childhood, especially after RSV bronchiolitis or rhinovirus bronchiolitis, is associated with the development of asthma later in life^5^,^14^. It is unknown whether the risk of asthma increases because of the bronchiolitis or whether a common genetic background underlies both diseases^15^. Polymorphisms in genes including *IL-10, IL-13, TLR4, VDR, CCR5*, and *ADAM33* have been associated with both RSV bronchiolitis and a risk of asthma^16^,^17^. This may support a genetic predisposition to both infant viral bronchiolitis and subsequent asthma. An increased risk of childhood-onset asthma after rhinovirus infection has been associated with 17q21 locus^18^.

Regarding bronchiolitis, genetic associations suggested in different studies need to be validated in further studies. A more comprehensive characterization of genetic determinants will lead to improved recognition of risk groups and will likely contribute to the development of new and more-specific treatments for respiratory infections. Therefore, the aim of the present study was to conduct a hypothesis-free genome-wide association study (GWAS) to identify genetic polymorphisms that predispose infants to viral bronchiolitis.

## Results

### Suggestive associations in GWAS of bronchiolitis and RSV bronchiolitis

We detected several suggestive association signals (*p* < 1 × 10^−5^) in the GWAS performed on 217 infants hospitalized for viral bronchiolitis and 778 controls. (Supplementary Figure S1). The stringent genome-wide significance level (*p* < 5 × 10^−8^) was not reached. Table 1 contains the results for the SNPs in the strongest suggestive GWA loci (*p* < 5 × 10^−6^). Cases were stratified according to presence of RSV, and RSV-positive bronchiolitis cases (*n* = 121) were tested against the same GWAS controls (“RSV GWAS”). Again, the signals did not reach the level of genome-wide significance (*p* < 5 × 10^−8^) (Supplementary Figure S2). Table 2 lists the strongest suggestive GWA loci (*p* < 5 × 10^−6^) in the RSV GWAS. Three of the best loci were shared between the bronchiolitis GWAS and the RSV GWAS. The shared loci were situated in the regions near the genes *VSTM4, C10orf71*, and *DRGX* in chromosome 10 and in the region near *LOC105375265* and *LOC105375266* in chromosome 7. Based on the results obtained from the bronchiolitis GWAS and the RSV GWAS, we selected one representative SNP for the replication phase from each of the best suggestive GWA region. All SNPs selected for replication genotyping and the respective results are presented in Supplementary Table S1.

### SNPs associated with RSV bronchiolitis in previous studies

We screened for SNPs that were reported to be associated with RSV bronchiolitis in previous studies and SNPs in the vicinity (within 20 kb) of such variants in our GWAS data. The exact SNPs reported in previous studies did not show associations or were not present in our GWAS data. The results for the variants near the reported bronchiolitis-associated SNPs are presented in Supplementary Table S2 (variants with *p* < 0.01 in the bronchiolitis GWAS or RSV GWAS are shown). The best SNP was rs56039226 in the *CX3CR1* intron (*p* = 5.7 × 10^−5^). This SNP is located 11 kb upstream of the nonsynonymous *CX3CR1* variant, rs3732378 (previously T280M). The A-allele–containing genotypes of rs3732378 were associated with RSV bronchiolitis severity in a previous study by Amanatidou *et al*.^19^. The exact SNP was present in our GWAS data, but it did not show an association (*p* = 0.095). However, the A-allele was also slightly more frequent in the GWAS cases compared to the controls (allele frequencies of 0.16 and 0.14 in cases and controls, respectively).

### SNPs associated with asthma in previous studies

When we screened SNPs that have been reported to be associated with asthma in the NHGRI-EBI GWAS Catalog (with *p* ≤ 10^−6^), no associations were seen in our data. The best SNP was rs12436663 (*p* = 5.8 × 10^−4^, OR = 1.96 for T allele in RSV GWAS) within *MRPP3* intron. The regions (20 kb) around the asthma-associated SNPs showed moderate signals in our data. The results are shown for variants with *p* ≤ 0.0001 in GWAS, RSV GWAS or GWAS with cases <1year (Supplementary Table S3). Many of the variants are known eQTLs. The variants showing strongest suggestive signals were in the area of *VCAN, HLA-DQA1*, and *NIN*. Those genes have various roles including cell adhesion, cytokine production, antigen binding and GTP binding^20^.

### Associations of rs269094, rs9591920, and rs1537091 with bronchiolitis in the replication populations

In the replication phase, we analyzed 77 of the most promising SNPs based on the results of the GWA analysis in an independent population collected in the Netherlands (416 cases and 432 controls). The suggestive associations of three of the SNPs (rs269094, rs9591920, and rs1537091) with bronchiolitis were nominally replicated (*p* < 0.05), and meta-analysis for these SNPs yielded lower *p* values compared to the original GWAS (Table 3). The results were similar for the RSV GWAS and GWAS of infants aged <1 year. The results for the SNPs with a *p* value <0.1 in the Dutch population and ORs in the same direction in the GWAS subsets, and meta-analyses, are shown in Supplementary Table S4.

To identify genes for which expression is potentially affected by our candidate SNPs (p value < 0.05 in the Dutch replicate), we queried genotype-tissue expression (GTEx) data^21^. Rs269094 was previously identified as an eQTL for *KCND3* in spleen (*p* = 7.1 × 10^−10^); the C-allele is associated with higher expression. Another correlated SNP in the same region (rs269101, r^2^ = 0.42 in 1000 genomes phase3 Finnish individuals) showed a trend toward association in our GWAS (*p* = 4.2 × 10^−5^) and in the replicate (*p* = 0.054), further supporting the result. By exploring the NHGRI-EBI GWAS Catalog, we also discovered that variants in or near *KCND3* have been associated with diisocyanite-induced asthma and the cytokine response to smallpox^22^. The two other candidate SNPs were not identified as known eQTLs.

We further screened the three SNPs (with p < 0.05 in the Dutch population) in the Finnish case data collected in Turku, using the NordicDB population controls as a reference (Supplementary Table S5). Rs9591920 showed an association (*p* = 0.042, OR = 1.5). The allele frequencies of the two other SNPs, rs269094 and rs1537091, did not differ between cases and controls in this analysis.

## Discussion

In the present study, we identified several suggestive GWA loci associated with viral bronchiolitis. Three of the SNPs selected for replication genotyping showed nominal associations (p < 0.05) also in an external replication population. Of those variants, rs269094 is a known eQTL for *KCND3*, which has been linked to diverse functions including regulation of neurotransmitter release and heart rate^20^. More recently, the gene has also been associated with diisocyanite-induced occupational asthma and cytokine response in smallpox vaccine recipients^23^,^24^. At the protein level, KCND3 interacts with another membrane protein, DPP10, of which genetic variants have been associated with conditions including asthma^20^,^25^. The association of another SNP with p < 0.05 in the Dutch population, rs9591920, was nominally replicated in the additional Finnish case set. The variant resides within an uncharacterized noncoding RNA (ncRNA) locus (*LOC105370220*)^20^. Thus, it is difficult to assign potential functions to this region. In general, intergenic or noncoding SNPs have been associated with many complex phenotypes in previous GWASs. The SNPs within ncRNA loci could affect either the expression or function of the RNA molecule, which may further regulate target gene expression^26^. The third variant showing nominal association in the Dutch cohort, rs1537091, is located in an intergenic region, nearest loci being long intergenic non-protein coding RNAs without known function. Further study is required to validate the associations of our candidate variants and study their potential mechanism in bronchiolitis susceptibility.

We detected only modest signals for the regions around SNPs reported by previous studies to be associated with bronchiolitis. This might be due to allele frequency or LD structure differences among study populations, false discoveries by chance due to limited population size or gene-environment interactions. The confounding effect of population stratification is difficult to take into account in candidate gene studies; in the present study, we could account for the slight structure caused by differing genetic origins by exploiting the genome-wide data and principal components and thus reduce the risk of false-positive findings. Further, we could exclude population outliers and close relatives based on information provided by genome-wide sampling. In our data, the best variant near the SNP that was previously reported to be associated with bronchiolitis was in *CX3CR1* intron (*p* = 5.7 × 10^−5^).

Due to suggested shared genetic contribution among bronchiolitis and asthma, variants that have been associated with asthma in previous studies, and regions (20 kb) around the variants, were screened in our data. Moderate signals (p < 0.0001) were detected in variants within genes including *VCAN* and *HLA-DQA1. HLA-DQ* region has been associated with different types of asthma in several studies^27^, and an association of *VCAN* with asthma was discovered more recently^28^. Asthma clusters in families and likely has a strong genetic component, with heritability estimates as high as 60%^29^. Hundreds of genetic polymorphisms have been associated with different asthma phenotypes in past genetic studies. However, asthma is a complex and heterogeneous disease which is likely contributed by a combination of many genes in interaction with environmental factors^30^. Thus, the potential roles of the suggested genes in LRI susceptibility, and an overlap with biological pathways related to asthma, warrant further study.

The present study had several limitations. The sample size was relatively small, and thus especially the stratified GWAS subset analyses might have been underpowered. In the discovery GWAS, the best suggestive association signals, that were almost genome-wide significant, were seen in the area of *LOC105375265* on chromosome 7 and in the area of *VSTM4* and *DRGX* on chromosome 10. The signals in these regions were identified both in the GWAS and the RSV GWAS but not in the Dutch replication population. Despite the lack of replication, these signals might well reflect true associations in the Finnish population.

There is no worldwide consensus on treatment and diagnosis of bronchiolitis. The American Academy of Pediatrics defines 24 months as an upper age limit of bronchiolitis^2^,^31^. In Finland and Sweden, clinicians have begun to consider bronchiolitis to be mainly a disease of infants under 12 months of age^32^. In our GWAS, most of the cases were <12 months of age at the time of the diagnosis. Moreover, for the most part the results for the GWAS <1 year were similar to those of the main GWAS. However, the differing clinical definitions of included bronchiolitis cases were one limitation of the current study. Further limitation was the differing proportion of RSV positive samples between the GWAS and the replication populations. In studies to come, selecting bronchiolitis study cases according to more specified criteria will likely help detecting greater effects and susceptibility genes associated with specific phenotypes.

The present GWAS identified several variants that were suggestively associated with bronchiolitis. Although no significant associations were detected, our study provides the first genome-wide examination of bronchiolitis and a large body of data that may benefit future studies on complex genetic determinants and biological pathways that cause variation in bronchiolitis susceptibility. Further replication and functional validation is needed to verify and uncover the potential roles of our candidate SNPs. The relationship between infant viral bronchiolitis and asthma, and a plausible genetic overlap of the two diseases, remain an important area of study.

## Materials and Methods

### Study design and populations

**Figure 1:**
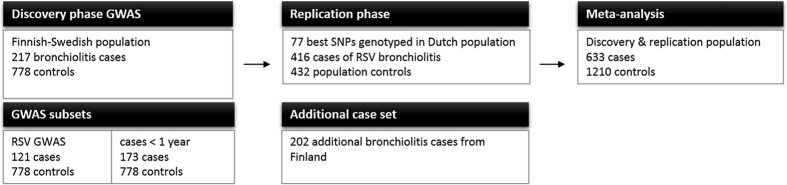
Flow chart of study design and populations. Primary endpoint studied in GWAS was viral bronchiolitis. Cases were further stratified into subsets according to presence of RSV and age. Stratified phenotypes studied were bronchiolitis caused by respiratory syncytial virus (“RSV GWAS”) and bronchiolitis in infants <12 months of age.

The study populations comprised a total of 2045 study subjects consisting of 835 bronchiolitis cases and 1210 controls (974 females and 1071 males). The study was approved by the ethics committees of Tampere University Hospital and Kuopio University Hospital, Finland, and the regional ethics committee of Gothenburg, Sweden. The study was carried out in accordance with the approved guidelines of the WMA Declaration of Helsinki. Informed consents were obtained from the study subjects or provided by their parents. The main elements of the study were: (1) a GWAS in a Finnish–Swedish discovery population of 217 cases and 778 controls, (2) an analysis of the most promising variants in a Dutch replication population of 416 cases and 432 controls, and (3) a further examination of the most promising variants in an additional Finnish set of 202 cases. Moreover, GWAS cases were stratified according to age or presence of RSV and the subgroups of RSV positive cases (“RSV GWAS”) and those of <12 months of age were studied separately (Fig. 1).

The inclusion criteria for all the affected children were (1) clinically diagnosed bronchiolitis, (2) an age <24 months at the time of diagnosis and (3) native Finnish, Swedish or Dutch ethnicity. Bronchiolitis was defined as the presence of LRI and respiratory symptoms typical of bronchiolitis, including wheezing and labored breathing^2^. RSV infection was tested by antigen immunofluorescent or radioimmuno assays, viral isolation, PCR of nasopharyngeal cells or by serum antibody assays.

Overall, RSV was detected in 72% of the cases, and >80% were under 12 months of age. Characteristics of bronchiolitis cases in GWAS discovery cohorts and validation cohorts are presented in Tables 4 and 5. The populations are further described in the Supplementary Material. Briefly, the Finnish individuals (*n* = 936) in the GWAS discovery population originated from Kuopio (64 cases, 64 controls), Tampere (124 cases), and Helsinki/Kuopio (684 controls) regions^33^,^34^,^35^,^36^. A subset of the GWAS population was collected in Gothenburg, Sweden (29 cases, 30 controls)^37^. Replication cohorts originated from The Netherlands (*n* = 848) and Finland (*n* = 202)^10^,^38^,^39^.

### DNA sample preparation and genotyping

Genomic DNA was obtained from whole blood samples, buccal swabs, or buffy coats. DNA from blood samples collected from the GWAS subjects was extracted with the UltraClean^®^ Blood DNA Isolation Kit (MO BIO Laboratories, Inc., Carlsbad, CA, USA). DNA from infants in the Dutch replication cohort was isolated from blood samples or buccal swabs with the QIAamp DNA Blood Kit (Qiagen, Hilden, Germany). DNA extraction from buffy coats collected from the Dutch population controls was done by digestion with proteinase K followed by salting out with potassium acetate and chloroform–isoamyl alcohol extraction.

Genome-wide genotyping was performed with the Human Genotyping Array Kits (Illumina, San Diego, CA, USA. (Supplementary Table S6). The Technology Centre at the Institute for Molecular Medicine Finland (FIMM), University of Helsinki, did the genotyping. FIMM also performed the targeted SNP genotyping in the replication phase with the Sequenom iPLEX Gold assay (San Diego, CA, USA). NordicDB controls are described in detail in Bilguvar *et al*.^40^.

### Quality control and imputation of the genome-wide data

After quality control (QC), 995 study subjects were available for the analyses: 464 male subjects and 531 female subjects/217 cases and 778 controls. There were 79,563 autosomal SNPs with a MAF of >0.01. The data was prephased with SHAPEIT2, followed by genotype imputation (i.e., statistical prediction of missing genotypes) with IMPUTE2^41^,^42^. More detailed description of the QC and imputation can be found in the online Supplementary Material. After imputation, the amount of SNPs (MAF >0.01) was 5,304,323.

### Selection of SNPs for replication analysis

Seventy-seven SNPs were genotyped in the Dutch replication population. Because the stringent genome-wide significance level (*p* < 5 × 10^−8^) was not reached in the discovery GWAS, less-strict criteria were applied for the selection of SNPs. *p* values of <5 × 10^−4^ and <1 × 10^−4^ for genotyped and imputed SNPs, respectively, were used as threshold values for suggestive associations. For imputed SNPs, linkage disequilibrium (LD) was taken into account by selecting relatively independent (r^2^ < 0.5) SNPs from each suggestive GWA locus. Sporadic imputed SNPs were excluded. Provided that a suggestive GWA locus had several variants with similar *p* values, the best SNP was chosen based on predicted or empirical regulatory functions (including eQTLs, promoter activity, and protein binding sites) by exploring annotation databases, including HaploReg v. 4.1, GTEx, and dbSNP^21^,^43^,^44^. We did not choose low-frequency SNPs (MAF <0.02 in cases or controls) in the main GWAS population to avoid genotyping monomorphic SNPs in the Dutch population. We tested the homogeneity of odds ratios among the GWAS subpopulations according to city of origin with the Breslow–Day test and excluded variants with *p* values of <0.01.

### Statistical analysis

#### Principal component analysis (PCA)

PCA with PLINK1.9 was performed on nonimputed LD-pruned GWAS data to delineate components explaining possible remaining population structure. The adequate number of components was evaluated based on genome-wide inflation factor lambda (λ), eigenvalues, and inspection of covariate significances in logistic regression. Three leading components were incorporated into subsequent association analyses.

#### SNP association analysis

Allele frequency differences were assessed with logistic regression under an additive model, with gender and three principal components as covariates. Analyses were performed with PLINK 1.9^45^. Lambda values were close to 1, indicating an adequately controlled population structure (λ = 1.004 for the genotyped population, λ = 1.06 for the imputed population). In the RSV GWAS, we studied a subset of cases positive for RSV infection (*n* = 121) against the GWAS controls (*n* = 778). Similarly, cases that were <12 months of age (173 cases, 778 controls) were tested as a group. Replication populations collected in the Netherlands and Turku, Finland, were studied with logistic regression analysis under an additive model, with gender as a covariate. Measures of basic QC, which entailed removing variants with MAF <0.01, genotyping rate <0.9, or deviation from HWE (*p* < 0.001), were performed. The replication case set of Turku was studied against the NordicDB population controls (*n* = 684). The discovery and the Dutch replication population were studied by meta-analysis under a fixed-effects model to estimate their combined effect. Confidence intervals for odds ratios of meta-analysis were calculated with a meta package in R^46^,^47^. Finally, SNPs that were reported in the literature to be associated with RSV bronchiolitis or asthma, or variants within 20 kb of such SNPs, were screened in our GWAS data.

## Additional Information

**How to cite this article**: Pasanen, A. *et al*. Genome-Wide Association Study of Polymorphisms Predisposing to Bronchiolitis. *Sci. Rep.* **7**, 41653; doi: 10.1038/srep41653 (2017).

**Publisher's note:** Springer Nature remains neutral with regard to jurisdictional claims in published maps and institutional affiliations.

## Supplementary Material

Supplementary Material

## Figures and Tables

**Table 1:** Strongest suggestive GWA loci (*p* < 5 × 10^−6^) identified by bronchiolitis GWAS and SNPs selected for replication genotyping from each locus.

Suggestive GWA locus╤					Selected SNP information							
Chr	RangeЖ	Span (kb)	SNPs	Lowest *p*	SNP name	LocationЖ	Genes**	A1	MAF		OR‡ (95% CI)	*p*
									Cases	Controls		
1	246851155..246968053	116	119	3.2 × 10^−6^	rs61202512	246860213	*SCCPDH, CNST*	A	0.464	0.327	1.67 (1.32–2.11)	1.6 × 10^−5^
2	235168096..235202022	33	29	2.8 × 10^−6^	rs11886348	235193436	*LOC105373933*	G	0.079	0.027	3.35 (2.02–5.55)	2.8 × 10^−6^
3	5377711..5570764	193	24	4.7 × 10^−6^	rs79062720	5442550	*MIR4790, EDEM1*	C	0.064	0.021	3.79 (2.14–6.70)	4.7 × 10^−6^
3	39318192..39593234	275	38	3.2 × 10^−6^	rs56039226	39318192	*CX3CR1*	A	0.041	0.011	4.43 (2.15–9.14)	5.7 × 10^−5^
3	107206802..107233889	27	6	3.0 × 10^−6^	rs6437736	107208688	*BBX, CCDC54*	A	0.366	0.491	0.58 (0.47–0.73)	3.0 × 10^−6^
3	153780474..153973898	193	9	5.0 × 10^−6^	rs36086140	153973898	*ARHGEF26*	T	0.093	0.037	2.86 (1.82–4.48)	5.0 × 10^−6^
4	24735463..24736258	0.8	2	2.6 × 10^−6^	rs4697072	24735463	*SOD3, CCDC149*	G	0.410	0.532	1.77 (1.40–2.25)	2.6 × 10^−6^
5	167829645..168048306	218	6	6.0 × 10^−7^	rs4976604	167849667	*WWC1*	C	0.064	0.021	3.80 (2.15–6.71)	4.3 × 10^−6^
7	46319511..46524796†	205	9	1.5 × 10^−7^	rs62452699	46492326	*LOC105375265, LOC105375266*	T	0.143	0.060	2.83 (1.92–4.17)	1.5 × 10^−7^
10	50452677..50460878†	8	10	4.9 × 10^−7^	rs17009617	50453933	*VSTM4, C10orf71*	A	0.090	0.032	3.39 (2.11–5.46)	4.9 × 10^−7^
10	50454331..50553085†	98	50	3.9 × 10^−7^	rs76728164	50465201	*C10orf71, DRGX*	T	0.088	0.032	2.93 (1.86–4.62)	3.7 × 10^−6^
10	50454331..50553085†	98	50	3.9 × 10^−7^	rs1001338	50498449	*C10orf71, DRGX*	G	0.086	0.031	3.10 (1.95–4–93)	1.7 × 10^−6^
10	128584158..128805211	221	12	2.1 × 10^−6^	rs111255454	128777619	*DOCK1*	G	0.065	0.021	3.90 (2.22–6.84)	2.1 × 10^−6^
12	17376616..17514609	137	37	3.8 × 10^−6^	rs2137526	17377995	*LOC105369677, LOC105369676*	T	0.233	0.148	1.98 (1.48–2.65)	3.8 × 10^−6^
13	59304857..59481192	176	40	2.0 × 10^−6^	rs9591920	59315119	*LOC105370220*	T	0.190	0.098	2.16 (1.57–2.97)	2.0 × 10^−6^
13	67847391..67877199	29	4	4.6 × 10^−7^	rs142670120	67850108	*PCDH9, LOC105370246*	DEL	0.099	0.037	3.23 (2.05–5.10)	4.6 × 10^−7^
16	73130946..73140092	9	5	2.4 × 10^−6^	rs76079505	73136490	*ZFHX3, C16orf47*	T	0.203	0.100	2.22 (1.59–3.09)	2.4 × 10^−6^
17	72973962..73220824	246	12	2.1 × 10^−6^	rs113409681	72973962	*HID1, CDR2L*	A	0.076	0.025	3.58 (2.11–6.05)	2.1 × 10^−6^
20	56486748..56527179	40	12	1.3 × 10^−6^	rs379083*	56527179	*MIR4532, LOC100129869*	T	0.112	0.043	2.82 (1.86–4.29)	1.3 × 10^−6^
21	42284256..42343366	59	52	2.6 × 10^−6^	rs117674297	42343366	*DSCAM, BACE2*	A	0.092	0.038	2.96 (1.88–4.65)	2.6 × 10^−6^

^╤^Each suggestive GWA locus comprises several SNPs in LD (r^2^ > 0.5, *p* < 0.01). Loci were formed with default options of LD-based result clumping (PLINK 1.9). We selected one variant for replication genotyping from each locus. Signals in sporadic imputed variants (not shown) were considered unreliable and not chosen for replication genotyping. Variants with a minor allele frequency of <0.02 within either cases or controls were not chosen for replication genotyping.

^Ж^Range and location refer to human genome build 37 (GRCh37/hg19) coordinates.

^**^Corresponding locus shown for variants within genes; two nearest loci shown for intergenic SNPs.

^‡^OR for A1 allele under additive model in logistic regression, with sex and three principal components as covariates.

^†^Suggestive GWA loci shared between GWAS and RSV GWAS (of loci with *p* < 5 × 10^−6^).

GWA, genome-wide association; SNP, single nucleotide polymorphism; Chr, chromosome; MAF, minor allele frequency; OR, odds ratio; CI, confidence interval; LD, linkage disequilibrium.

**Table 2:** Strongest suggestive GWA loci (*p* < 5 × 10^−6^) from RSV bronchiolitis GWAS and SNPs selected for replication genotyping from each locus.

Suggestive RSV GWA locus╤					Selected SNP information							
Chr	RangeЖ	Span (kb)	SNPs	Lowest *p*	SNP name	LocationЖ	Genes**	A1	MAF		OR‡ (95% CI)	*p*
									Cases	Controls		
1	153512691..153996110	483	6	5.2 × 10^−7^	rs75940909	153932075	*SLC39A1*	A	0.063	0.011	5.58 (2.69–11.93)	9.2 × 10^−6^
1	154011426..154260767	249	4	1.1 × 10^−6^	rs186841738	154187935	*C1orf43*	A	0.062	0.009	6.73 (3.02–15.01)	3.1 × 10^−6^
2	36592600..36617435	24	2	2.4 × 10^−6^	rs112913823	36617435	*CRIM1*	G	0.086	0.025	4.38 (2.37–8.09)	2.4 × 10^−6^
3	185675973..185677613	1	9	4.9 × 10^−6^	rs6444089	185676350	*TRA2B, ETV5*	G	0.277	0.410	0.48 (0.34–0.66)	8.7 × 10^−6^
3	185675973..185677613	1	9	4.9 × 10^−6^	rs55675198	185677613	*TRA2B, ETV5*	G	0.252	0.391	0.46 (0.33–0.64)	6.2 × 10^−6^
4	32653789..32869901	216	52	4.7 × 10^−6^	rs4547837	32688632	*LOC107986223, LOC101927363*	T	0.054	0.010	6.55 (2.93–14.65)	4.7 × 10^−6^
7	46319511..46524796^†^	205	9	6.9 × 10^−7^	rs62452699	46492326	*LOC105375265, LOC105375266*	T	0.149	0.061	3.37 (2.09–5.45)	6.9 × 10^−7^
7	148765688..148959272	193	21	5.5 × 10^−7^	rs139743624	148870713	*ZNF398*	A	0.051	0.010	6.51 (2.86–14.86)	8.5 × 10^−6^
10	50452677..50460878†	8	10	3.1 × 10^−7^	rs17009617	50453933	*VSTM4, C10orf71*	A	0.111	0.032	4.16 (2.41–7.19)	3.1 × 10^−7^
10	50454331..50553085†	98	50	1.2 × 10^−7^	rs76728164	50465201	*C10orf71, DRGX*	T	0.106	0.033	3.46 (2.05–5.85)	3.6 × 10^−6^
10	50454331..50553085†	98	50	1.2 × 10^−7^	rs1001338	50498449	*C10orf71, DRGX*	G	0.112	0.031	3.95 (2.33–6.69)	3.6 × 10^−7^
15	39354517..39515134	160	46	6.5 × 10^−7^	rs75360543	39505815	*LOC105370777*	G	0.104	0.034	4.10 (2.35–7.14)	6.5 × 10^−7^

^╤^Each suggestive GWA locus comprises several SNPs in LD (r^2^ > 0.5, *p* < 0.01). Loci were formed with default options of LD-based result clumping (PLINK 1.9). We selected one variant for replication genotyping from each locus. Signals in sporadic imputed variants (not shown) were considered unreliable and not chosen for replication genotyping.

^Ж^Range and location refer to human genome build 37 (GRCh37/hg19) base pair positions.

^**^Corresponding locus shown for variants within genes; two nearest genes shown for intergenic SNPs.

^‡^OR for A1 allele under additive model in logistic regression, with sex and three principal components as covariates.

^†^Suggestive GWA loci shared between GWAS and RSV GWAS (of loci with *p* < 5 × 10^−6^).

GWA, genome-wide association; RSV, respiratory syncytial virus; SNP, single nucleotide polymorphism; Chr, chromosome; MAF, minor allele frequency; OR, odds ratio; CI, confidence interval; LD, linkage disequilibrium.

**Table 3:** SNPs that had a *p* value of <0.05 in the Dutch replicate, with ORs in the same direction compared to GWAS.

Variant details					GWAS		Replication		Meta-analysis§	
SNP	Chr	LocationЖ	Genes**	A1	OR‡ (95% CI)	*p*	OR╤ (95% CI)	*p*	OR (95% CI)	*p*
rs269094	1	112639174	*KCND3, LOC643355*	C	1.67 (1.30–2.15)	5.3 × 10^−5^	1.28 (1.03–1.60)	0.0292	1.44 (1.22–1.70)	1.5 × 10^−5^
rs9591920	13	59315119	*LOC105370220*	T	2.16 (1.57–2.97)	2.0 × 10^−6^	1.41 (1.05–1.88)	0.0227	1.71 (1.38–2.12)	9.8 × 10^−7^
rs1537091	21	29004226	*LOC105372763, LINC00113*	A	1.69 (1.34–2.14)	1.3 × 10^−5^	1.24 (1.01–1.52)	0.0408	1.42 (1.21–1.66)	1.1 × 10^−5^

^Ж^Location refers to human genome build 37 (GRCh37/hg19) coordinates.

^**^Corresponding locus shown for variants within genes; two nearest genes shown for intergenic SNPs.

^‡^OR for A1 allele under additive model in logistic regression, with sex and three principal components as covariates.

^╤^OR for A1 allele under additive model in logistic regression, with sex as a covariate.

^§^Meta-analysis performed under fixed-effects model for logistic regression result files of discovery and replication data.

SNP, single nucleotide polymorphism; GWAS, genome-wide association study; RSV, respiratory syncytial virus; OR, odds ratio; CI, confidence interval; Chr, chromosome.

**Table 4:** Characteristics of study cases in GWAS bronchiolitis populations.

	All		Kuopio, FIN		Tampere, FIN		Gothenburg, SWE	
	n	%	n	%	n	%	n	%
All cases	217	100%	64	100%	124	100%	29	100%
RSV positive	121	56%	21	33%	90	73%	10	36%
RSV negative	94	43%	41	64%	34	27%	19	64%
Age <12 months	173	80%	33	52%	124	100%	16	55%
Age >12 months	42	19%	29	45%	0	0%	13	45%
Males	114	53%	36	56%	62	50%	16	55%
Hospitalization rate		100%		100%		100%		100%

GWAS, genome-wide association study; FIN, Finland; SWE, Sweden.

**Table 5:** Characteristics of bronchiolitis cases in replication populations.

	Netherlands		Turku, Finland	
	n	%	n	%
All cases	416	100%	202	100%
RSV positive*	416	100%	62	31%
Males	237	60%	140	69%
Age <12 months		95%**		50%
Hospitalization rate		100%		90%

^*^Only RSV-positive bronchiolitis cases originally selected^10^.

^**^Cases were randomly acquired from a population of >95% of the cases <12 months of age^10^.
