# Supplementary material for: Genome-Wide Association Study of Polymorphisms Predisposing to Bronchiolitis
Source: Sci Rep. 2017 Jan 31;7:41653. doi: 10.1038/srep41653 (PMC5282585; doi:10.1038/srep41653)
Supplement: Supplementary Material [file srep41653-s1.pdf]

SUPPLEMENTARY MATERIAL

GENOME-WIDE ASSOCIATION STUDY OF POLYMORPHISMS PREDISPOSING TO BRONCHIOLITIS

Anu Pasanen[1*], Minna K. Karjalainen[1], Louis Bont[2], Eija Piippo-Savolainen[3], Marja Ruotsalainen[3], Emma Goksör[4], Kuldeep Kumawat[5], Hennie Hodemaekers[6], Kirsi Nuolivirta[7], Tuomas Jartti[8], Göran Wennergren[4], Mikko Hallman[1], Mika Rämet[1,9†], Matti Korppi[10†]

* **Correspondence**: Anu Pasanen, MSc, Department of Children and Adolescents, Institute of Clinical Medicine, University of Oulu, 90220 Oulu, Finland. Tel. +358 50 342 7253, Fax +358 8 315 5559, E-mail: anu.pasanen@oulu.fi

†These authors contributed equally to this work.

The enrollment of GWAS populations

The Finnish individuals (*n* = 936) in the GWAS discovery population originated from Kuopio (64 cases, 64 controls), Tampere (124 cases), and Helsinki/Kuopio (684 controls) regions. Cases from Kuopio comprised two prospective cohorts of term children admitted to Kuopio university hospital with bronchiolitis in 1981–1982 and 1992–1993 [1, 2]. Age matched controls (+/- two months) were collected for genetic analysis. Chronic pulmonary disease (including asthma) was an exclusion criterion for cases. For controls, exclusion criteria were wheezing or hospitalization before the age of 24 months. GWAS subjects from Tampere were healthy full-term infants less than 12 months of age hospitalized for bronchiolitis at Tampere university hospital [3]. The enrollment periods were between December 2001 and May 2002, and between October 2002 and May 2004. The controls from the Helsinki/Kuopio region were anonymous population controls obtained through the Nordic Control database (NordicDB) [4, 5]. The controls were selected from the database based on a city of origin. Remaining outliers were excluded based on genome-wide identity-by-state (IBS) information. A Swedish subset of the GWAS population (29 cases and 30 controls) comprised a prospective cohort of children who were hospitalized with a first wheezing episode (wheezing bronchitis/acute bronchiolitis) [6, 7] and age-matched controls (+/- two months). Hospitalizations occurred between March 1984 and November 1985.

Replication populations

Dutch population (*n* = 848) included previously healthy, native white Dutch children (*n* = 416) who were hospitalized for RSV-associated LRI during 1992–2006 and unselected controls born in the Netherlands from a Dutch health examination survey, the Regenboog study (*n* = 432) [8, 9]. Children with previous airway morbidity, wheeze or airway medication were excluded. A case set for further replication comprised 202 bronchiolitis cases aged 3–23 months collected in Turku, Finland, between September 2000 and May 2002, and between June 2007 and March 2010 (Vinku and Vinku2 studies) [10, 11]. These

cases were tested against the NordicDB population controls. Exclusion criteria included more than one previous wheezing attack, inhaled or systemic corticosteroids and chronic nonatopic disease.

Quality control and imputation of the genome-wide data

We performed quality control (QC) of the genome-wide SNP data with PLINK 1.90 beta ("PLINK 1.9") [12, 13] and removed samples with a genotype call rate of <97%. Variants with a minor allele frequency (MAF) of <0.01, a genotyping rate of <0.9 or deviation from Hardy–Weinberg equilibrium (HWE, $p < 0.001$) were excluded. Based on pairwise identity-by-state (IBS) distances and identity-by-descent (IBD) similarities, fourteen samples were excluded as population outliers or to avoid confounding by cryptic relatedness (pihat cut-off 0.15). After QC, 995 study subjects were available for the analyses: 464 male subjects and 531 female subjects/217 cases and 778 controls. There were 79,563 autosomal SNPs with a MAF of >0.01.

Genome-wide data was prephased with SHAPEIT2, followed by genotype imputation (i.e., statistical prediction of missing genotypes) of autosomal chromosomes with IMPUTE2 [14, 15]. The reference panel used for prephasing and imputation was the 1,000 Genomes Phase 3 integrated variant set (release in NCBI build 37 (hg19) coordinates, 12 October 2014). Populations from the areas of Kuopio and Gothenburg, which were genotyped in the same platform, were imputed together first, and the population from Tampere was imputed together with the NordicDB controls later. We combined the two imputed data sets and implemented basic QC measures, which entailed removing variants with MAF < 0.01, genotyping rate < 0.9, or deviation from HWE ($p < 0.001$). An imputation info score of 0.7 was used as the cut-off value for excluding poorly imputed variants. The overall proportion of genotypes with non-missing data was 0.97. There were 5,304,323 SNPs post-imputation and QC.

References

1. Reijonen, T., Korppi, M., Pitkakangas, S., Tenhola, S. & Remes, K. The clinical efficacy of nebulized racemic epinephrine and albuterol in acute bronchiolitis. *Arch. Pediatr. Adolesc. Med.* **149**, 686-692 (1995).

2. Korppi, M., Halonen, P., Kleemola, M. & Launiala, K. Viral findings in children under the age of two years with expiratory difficulties. *Acta Paediatr. Scand.* **75**, 457-464 (1986).

3. Koponen, P., Helminen, M., Paassilta, M., Luukkaala, T. & Korppi, M. Preschool asthma after bronchiolitis in infancy. *Eur. Respir. J.* **39**, 76-80 (2012).

4. Leu, M. *et al*. NordicDB: a Nordic pool and portal for genome-wide control data. *Eur. J. Hum. Genet.* **18**, 1322-1326 (2010).

5. Bilguvar, K. *et al*. Susceptibility loci for intracranial aneurysm in European and Japanese populations. *Nat. Genet.* **40**, 1472-1477 (2008).

6. Goksor, E. *et al*. High risk of adult asthma following severe wheezing in early life. *Pediatr. Pulmonol.* **50**, 789-797 (2015).

7. Wennergren, G. *et al*. Characteristics and prognosis of hospital-treated obstructive bronchitis in children aged less than two years. *Acta Paediatr.* **81**, 40-45 (1992).

8. Viet AL , Kroesbergen HT , Verhoeff AP , Seidell JC , Otten F , Veldhuizen H van. Risk Factors and Health in the Netherlands, a Survey by Municipal Public; Regional results. RIVM Report 260854005. (2004).

9. Janssen, R. *et al*. Genetic susceptibility to respiratory syncytial virus bronchiolitis is predominantly associated with innate immune genes. *J. Infect. Dis.* **196**, 826-834 (2007).

10. Lehtinen, P. *et al*. Prednisolone reduces recurrent wheezing after a first wheezing episode associated with rhinovirus infection or eczema. *J. Allergy Clin. Immunol.* **119**, 570-575 (2007).

11. Jartti, T. *et al*. Short- and long-term efficacy of prednisolone for first acute rhinovirus-induced wheezing episode. *J. Allergy Clin. Immunol.* **135**, 691-8.e9 (2015).

12. Chang, C. C. *et al*. Second-generation PLINK: rising to the challenge of larger and richer datasets. *Gigascience* **4**, 7-015-0047-8. eCollection 2015 (2015).

13. Chang, C. C. & Purcell, S. PLINK 1.9. https://www.cog-genomics.org/plink2.

14. O'Connell, J. *et al*. A General Approach for Haplotype Phasing across the Full Spectrum of Relatedness. *PLoS Genet.* **10**, e1004234. doi:10.1371/journal.pgen.1004234 (2014).

15. Howie, B. N., Donnelly, P. & Marchini, J. A flexible and accurate genotype imputation method for the next generation of genome-wide association studies. *PLoS Genet.* **5**, e1000529 (2009).

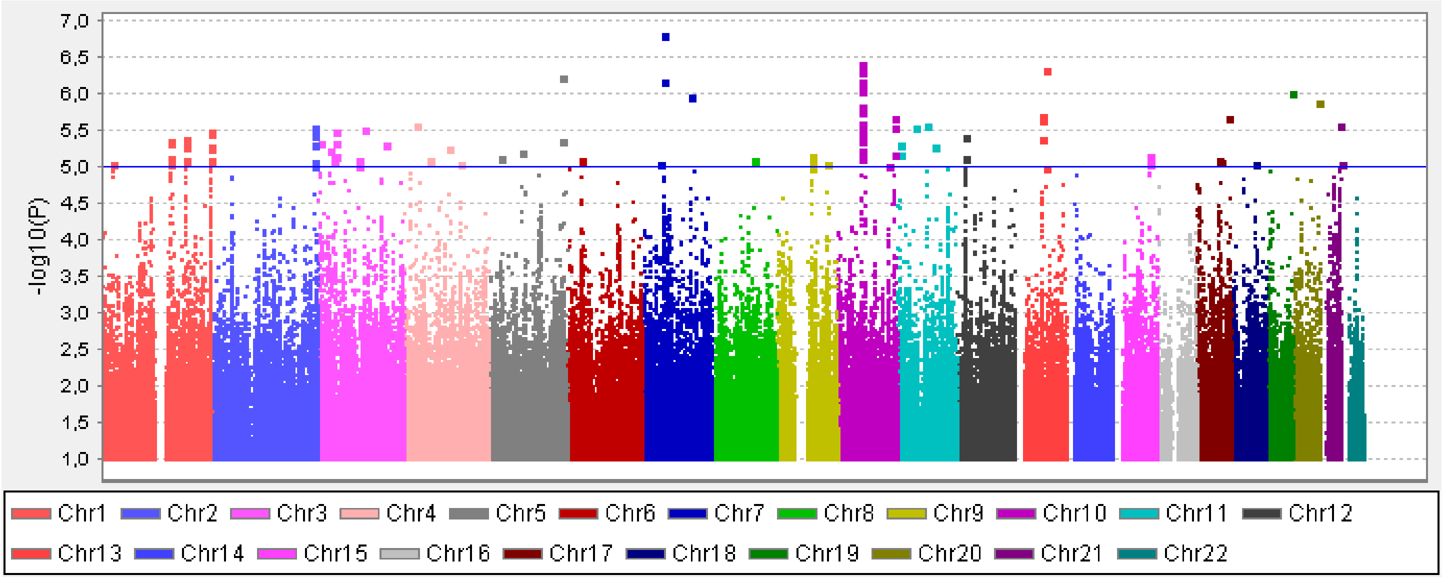

Supplementary Figure S1. Manhattan plot showing bronchiolitis GWAS results for the discovery population of 217 cases and 778 controls analyzed with logistic regression under an additive model. X-axis represents the genome in physical order, y-axis shows −log10 *p* values for all SNPs (starting from 0.01). Blue line represents the threshold of suggestive associations ($p < 1 \times 10^{-5}$). The stringent level of genome-wide significance ($p < 5 \times 10^{-8}$) was not reached. The strongest suggestive signals were detected in the areas of *VSTM4*, *C10rf71*, and *DRGX* in chromosome 10 and *LOC105375265* and *LOC105375266* in chromosome 7.

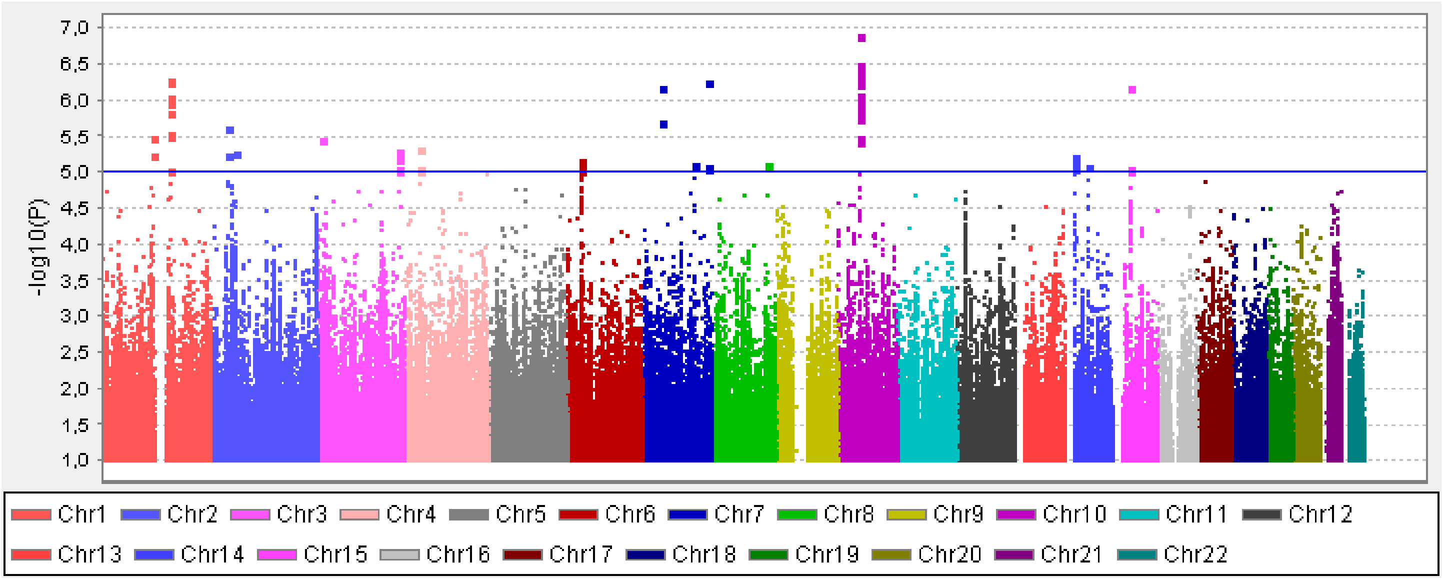

Supplementary Figure S2. Manhattan plot showing results of the RSV bronchiolitis GWAS (121 cases, 778 controls). X-axis shows chromosomes 1–22, y-axis shows −log10 *p* values for all SNPs (starting from 0.01). The stringent level of genome-wide significance ($p < 5 \times 10^{-8}$) was not reached. Blue line represents the threshold of suggestive associations ($p < 1 \times 10^{-5}$). The strongest suggestive signal was detected in the area near genes *VSTM4*, *C10rf71*, and *DRGX* in chromosome 10. The best signal in chromosome 10 was shared between bronchiolitis GWAS and RSV bronchiolitis GWAS.

Supplementary Table S1. SNPs that that were chosen for replication genotyping in an independent Dutch population (p =<$5 \times 10^{-4} - 1.2 \times 10^{-7}$ in the whole GWAS population or in the RSV subset of GWAS). Blue shading denotes SNPs with ORs in the same direction in the discovery and replication populations.

Variant information					Bronchiolitis GWAS		RSV bronchiolitis GWAS		Replication genotyping		
Chr	SNP	LocationӁ	Genes**	A1	OR‡	p	OR‡	p	A1	OR₸	p
1	rs75508464	22288656	*CELA3B, HSPG2*	C	2.27	9.0×10^{-6}	2.21	3.7×10^{-4}	C	0.80	0.242
1	rs522468†	36653677	*MAP7D1, TRAPPC3*	T	1.33	0.023	1.78	2.1×10^{-4}	-	-	-
1	rs35665787†	43233988	*C1orf50*	G	1.77	0.013	2.88	3.4×10^{-5}	-	-	-
1	rs269101	112628860	*LOC643355, KCND3*	T	1.61	4.2×10^{-5}	1.70	3.0×10^{-4}	T	1.21	0.054
1	rs269094	112639174	*LOC643355, KCND3*	C	1.67	5.3×10^{-5}	1.81	1.9×10^{-4}	C	1.28	0.029
1	rs59449379	117480316	*PTGFRN*	A	1.85	6.8×10^{-5}	2.36	3.2×10^{-6}	A	0.69	0.018
1	rs75940909	153932075	*SLC39A1*	A	3.97	1.0×10^{-4}	5.58	9.2×10^{-6}	A	0.82	0.600
1	rs186841738†	154187935	*C1orf43*	A	4.83	2.6×10^{-5}	6.73	3.1×10^{-6}	-	-	-
1	rs35835690†	189820786	*LOC107985456, LOC105371657*	DEL	2.80	1.1×10^{-5}	2.41	0.0033	-	-	-
1	rs61202512	246860213	*LOC102724382, SCCPDH*	A	1.67	1.6×10^{-5}	1.49	0.0077	A	1.00	0.971
1	rs3007311†	246866902	*LOC102724382, SCCPDH*	T	1.69	1.1×10^{-5}	1.62	0.0014	-	-	-
2	rs112913823	36617435	*CRIM1*	G	2.65	4.8×10^{-5}	4.38	2.4×10^{-6}	G	1.15	0.483
2	rs17749106	46719922	*ATP6V1E2*	C	1.66	1.4×10^{-5}	1.60	0.0016	C	1.01	0.929
2	rs12712976	46786491	*RHOQ*	A	0.59	8.2×10^{-5}	0.46	1.6×10^{-5}	A	1.01	0.905
2	rs782605╥	55888413	*PNPT1*	G	2.04	0.0061	3.61	5.2×10^{-6}	-	-	-
2	rs706551	55903620	*PNPT1*	G	1.67	0.028	2.60	1.6×10^{-4}	G	1.40	0.125
2	rs774503	155399735	*LOC105373693*	T	1.73	2.7×10^{-5}	1.74	6.5×10^{-4}	T	0.93	0.507
2	rs11886348	235193436	*LOC105373933*	G	3.35	2.8×10^{-6}	3.22	1.6×10^{-4}	G	1.08	0.791
2	rs6437298	241120401	*OTOS, COPS9*	G	1.70	2.0×10^{-4}	2.12	2.3×10^{-5}	G	0.82	0.093
3	rs79062720*	5442550	*MIR4790, EDEM1*	C	3.79	4.7×10^{-6}	4.04	8.3×10^{-5}	-	-	-
3	rs9880307†	16974610	*PLCL2*	A	0.69	0.0022	0.53	5.9×10^{-5}	-	-	-
3	rs6775709†	17114036	*PLCL2*	C	0.73	0.0081	0.53	5.1×10^{-5}	-	-	-
3	rs4685426	17119238	*PLCL2*	G	0.75	0.015	0.54	1.0×10^{-4}	G	0.98	0.845
3	rs35439956	25953521	*LINC00692, OXSM*	G	4.28	1.6×10^{-5}	3.92	6.5×10^{-4}	G	1.07	0.811
3	rs7638123†	36363607	*STAC, DCLK3*	A	2.06	7.4×10^{-6}	-	-	-	-	-
3	rs2127231	36391997	*STAC, DCLK3*	A	2.06	1.6×10^{-5}	2.43	2.4×10^{-5}	A	1.03	0.860
3	rs56039226†	39318192	*CX3CR1*	A	4.43	5.7×10^{-5}	4.17	9.8×10^{-4}	-	-	-
3	rs6437736	107208688	*BBX, LOC101929607*	A	0.58	3.0×10^{-6}	0.63	0.0016	A	1.18	0.088
3	rs13079196	143197761	*SLC9A9*	G	2.20	1.4×10^{-5}	2.45	6.4×10^{-5}	G	0.76	0.121
3	rs35443555	143359156	*SLC9A9*	T	2.20	4.2×10^{-4}	3.04	1.8×10^{-5}	T	0.89	0.643
3	rs36086140	153973898	*ARHGEF26*	T	2.86	5.0×10^{-6}	2.85	2.4×10^{-4}	T	0.91	0.682
3	rs6444089	185676350	*TRA2B, ETV5*	G	0.66	6.3×10^{-4}	0.48	8.7×10^{-6}	G	1.02	0.877
3	rs55675198	185677615	*TRA2B, ETV5*	DEL	0.66	8.4×10^{-4}	0.46	6.2×10^{-6}	DEL	1.02	0.822
4	rs4697072	24735463	*SOD3, CCDC149*	G	0.56	2.6×10^{-6}	0.56	1.9×10^{-4}	G	1.08	0.451
4	rs4547837	32688632	*LOC107986223, LOC101927363*	T	4.96	1.6×10^{-5}	6.55	4.7×10^{-6}	T	0.97	0.934
4	rs79316039†	76644558	*G3BP2*	A	2.68	3.7×10^{-5}	3.19	5.9×10^{-5}	-	-	-
4	rs143848875†	89178625	*PPM1K, ABCG2*	C	2.96	2.5×10^{-4}	4.05	4.7×10^{-5}	-	-	-
4	rs7682161	89191346	*PPM1K*	C	3.08	1.6×10^{-4}	4.17	3.5×10^{-5}	C	0.86	0.526
4	rs1517945	137938336	*LOC105377441*	C	0.68	0.0012	0.58	3.5×10^{-4}	C	0.89	0.216
5	rs13182546	82831359	*VCAN*	G	1.81	2.4×10^{-5}	1.90	2.7×10^{-4}	G	1.03	0.775
5	rs529998	82841764	*VCAN*	T	1.47	0.0010	1.95	1.7×10^{-5}	T	1.09	0.391

5	rs4976604	167849667	*WWC1*	C	3.80	4.3×10^{-6}	3.49	4.5×10^{-4}	C	0.75	0.244
6	rs9502516	6905830	*RP3-429O6.1*	A	1.35	0.012	1.77	2.0×10^{-4}	A	0.98	0.861
6	rs2844575†	31334945	*HLA-B, MIR6891*	C	1.56	1.5×10^{-4}	1.77	1.7×10^{-4}	-	-	-
6	rs1130153†	32610947	*HLA-DQA1*	C	2.72	7.7×10^{-6}	1.73	0.073	-	-	-
6	rs28633411†	32613362	*HLA-DQA1*	A	2.58	2.9×10^{-5}	1.63	0.120	-	-	-
6	rs210183	33486841	*BAK1, RN7SL26P-201*	G	1.40	0.011	2.06	8.1×10^{-6}	G	0.87	0.182
6	rs210184†	33486954	*BAK1, RN7SL26P-201*	G	-	-	2.06	8.1×10^{-6}	-	-	-
6	rs210196	33507723	*BAK1, RN7SL26P-201*	G	1.40	0.011	2.03	1.1×10^{-5}	G	0.89	0.289
7	rs1525738	16889926	*AGR3, AGR2*	T	1.35	0.0083	1.68	3.7×10^{-4}	T	1.05	0.638
7	rs10253036	46038606	*LOC102723446*	T	2.06	1.6×10^{-5}	1.90	0.0023	T	0.77	0.086
7	rs62452699	46492326	*LOC105375265, LOC105375266*	T	2.83	1.5×10^{-7}	3.37	6.9×10^{-7}	T	0.82	0.234
7	rs31674†<br>*tSNP*: *rs31672*	87068464	*ABCB4*	A	1.67	1.1×10^{-4}	1.85	1.9×10^{-4}	*tSNP*<br>C	*tSNP*<br>0.89	*tSNP*<br>0.321
7	rs144739276*	120062433	*KCND2*	G	2.27	8.8×10^{-5}	2.86	2.8×10^{-5}	-	-	-
7	rs139743624	148870713	*ZNF398*	A	4.25	1.8×10^{-4}	6.51	8.5×10^{-6}	A	0.82	0.511
8	rs12541098	67336382	*RRS1-AS1*	T	0.64	2.0×10^{-4}	0.57	3.2×10^{-4}	T	0.88	0.221
8	rs11985337	82443387	*FABP12*	C	1.43	0.0040	2.00	6.1×10^{-6}	C	0.95	0.673
9	rs10738377	14757744	*FREM1*	A	0.62	8.9×10^{-4}	0.49	3.3×10^{-4}	A	0.82	0.066
9	rs10756611	14765611	*FREM1*	G	0.53	2.6×10^{-5}	0.43	4.1×10^{-5}	G	0.81	0.064
9	rs6560613	80340387	*GNAQ*	C	2.48	7.7×10^{-7}	2.42	6.8×10^{-4}	C	0.88	0.533
9	rs3824464	114690765	*UGCG*	G	1.52	2.7×10^{-4}	1.36	0.036	G	0.93	0.510
9	rs77887191*	118451733	*LOC105376234, LOC105376235*	G	2.44	0.0013	3.58	4.2×10^{-5}	-	-	-
10	rs1334893	27693305	*PTCHD3*	G	2.34	1.8×10^{-4}	3.11	0.0014	G	1.02	0.927
10	rs17009617	50453933	*VSTM4, C10orf71*	A	3.39	4.9×10^{-6}	4.16	3.1×10^{-7}	A	1.00	0.989
10	rs76728164	50465201	*C10orf71, DRGX*	T	2.93	3.7×10^{-6}	3.46	3.6×10^{-6}	T	1.33	0.233
10	rs1001338	50498449	*C10orf71, DRGX*	G	3.10	1.7×10^{-6}	3.89	3.6×10^{-7}	G	1.38	0.183
10	rs7099976	83865764	*NRG3*	A	1.62	2.8×10^{-4}	1.47	0.058	A	0.89	0.299
10	rs111255454*	128777619	*DOCK1*	G	3.90	2.1×10^{-6}	3.03	0.0030	-	-	-
11	rs9736426†	3597515	*LOC101927708, ART5*	G	1.63	2.1×10^{-4}	1.67	0.0019	-	-	-
11	rs75404994†	66612234	*RCE1*	T	2.23	1.2×10^{-5}	-	-	-	-	-
11	rs6589464	114811893	*ACA59, CADM1*	C	0.57	3.5×10^{-5}	0.58	0.0015	C	1.07	0.486
12	rs2137526†	17377995	*LOC105369677, LOC105369676*	T	1.98	3.8×10^{-6}	2.09	8.8×10^{-5}	-	-	-
12	rs73070967†	18375073	*RERGL*	T	1.67	2.1×10^{-4}	2.00	4.3×10^{-5}	-	-	-
12	rs7314145	18375513	*RERGL*	C	1.68	1.5×10^{-4}	2.02	2.3×10^{-5}	C	0.97	0.837
12	rs7314267	76291888	*PHLDA1, LOC105369846*	A	1.39	0.034	1.93	2.9×10^{-4}	A	0.94	0.739
13	rs9597824†	59313613	*LOC105370220*	G	2.16	2.0×10^{-6}	2.03	3.6×10^{-4}	-	-	-
13	rs9591920	59315119	*LOC105370220*	T	2.16	2.0×10^{-6}	2.03	3.5×10^{-4}	T	1.41	0.023
13	rs142670120	67850108	*PCDH9, LOC105370246*	DEL	3.23	4.6×10^{-7}	3.45	3.0×10^{-5}	DEL	1.14	0.408
13	rs35746848	99059276	*FARP1*	T	2.45	1.1×10^{-4}	3.00	9.5×10^{-5}	T	0.77	0.152
14	rs72686085	22121065	*TRA*	C	2.26	1.7×10^{-4}	3.81	6.2×10^{-6}	C	0.75	0.248
14	rs200994756†	22125609	*TRA*	C	2.23	0.0020	3.79	6.9×10^{-6}	-	-	-
14	rs7159936	22172775	*TRA*	A	2.10	0.0033	3.68	6.2×10^{-6}	A	0.85	0.453
14	rs72688054	22190160	*TRA*	C	2.11	0.0031	3.69	6.0×10^{-6}	C	0.84	0.403
14	rs7143959±	51245666	*NIN*	T	1.32	0.0160	1.87	2.0×10^{-5}	-	-	-
14	rs714421	71359585	*PCNX1, LOC105370557*	T	0.73	0.0053	0.58	2.6×10^{-4}	T	0.98	0.834
14	rs8007693	80561521	*DIO2, LOC105370591*	C	1.43	0.0013	1.66	3.9×10^{-4}	C	1.07	0.483
15	rs114721875╥	39370956	*LOC105370777*	A	2.04	0.0029	3.21	3.5×10^{-5}	-	-	-

15	rs75360543	39505815	*LOC105370777*	G	2.46	3.0×10^{-4}	4.10	6.5×10^{-7}	G	0.94	0.790
15	rs664936	59064786	*FAM63B*	G	0.62	4.4×10^{-4}	0.69	0.030	G	1.07	0.565
15	rs4777160	64142539	*HERC1, MIR422A*	C	0.64	1.5×10^{-4}	0.54	5.9×10^{-5}	T	0.90	0.289
15	rs192701158†	82394155	*LINC01583, EFL1*	T	2.31	7.0×10^{-6}	1.79	0.0190	-	-	-
15	rs950419	82398217	*LINC01583, EFL1*	G	2.26	1.3×10^{-5}	1.87	0.0096	G	1.01	0.960
15	rs187513321†	82398874	*LINC01583, EFL1*	T	2.31	8.1×10^{-6}	1.91	0.0077	-	-	-
16	rs1507010	7657373	*RBFOX1*	A	1.58	6.1×10^{-5}	1.79	7.7×10-5	A	0.95	0.598
16	rs11639567	54415702	*LOC100996345, IRX3*	G	1.40	0.0057	1.74	2.6×10^{-4}	G	1.11	0.292
16	rs76079505†	73136490	*ZFHX3, C16orf47*	T	2.22	2.4×10^{-6}	-	-	-	-	-
16	rs2240245	74758984	*FA2H*	A	1.84	1.0×10^{-4}	2.21	3.3×10^{-5}	A	0.91	0.475
17	rs35739738╥	1023740	*ABR*	T	3.11	1.9×10^{-5}	3.19	2.9×10^{-4}	-	-	-
17	rs35883991†	1023778	*ABR*	T	3.13	1.7×10^{-5}	3.28	2.2×10^{-4}	-	-	-
17	rs62068097	26064677	*NOS2, LGALS9*	A	2.82	0.0013	4.19	6.6×10^{-5}	A	1.07	0.788
17	rs113409681*	72973962	*HID1, CDR2L*	A	3.58	2.1×10^{-6}	3.24	5.2×10^{-4}	-	-	-
18	rs17446644	25694712	*CDH2*	A	2.84	1.5×10^{-5}	2.59	0.0020	A	0.92	0.719
19	rs2240702	4159873	*CREB3L3*	G	1.82	1.3×10^{-4}	1.96	5.0×10^{-4}	G	1.03	0.857
19	rs62124697†	8141455	*FBN3*	A	5.88	6.9×10^{-5}	8.65	3.1×10^{-5}	-	-	-
20	rs144475389	17351353	*PCSK2*	T	3.09	2.8×10^{-5}	3.62	5.7×10^{-5}	T	1.17	0.591
20	rs379083*	56527179	*MIR4532, LOC100129869*	T	2.82	1.3×10^{-6}	-	-	-	-	-
21	rs242341	28966522	*LOC105372763, LINC00113*	T	0.71	0.0029	0.56	1.1×10^{-4}	T	0.89	0.230
21	rs1537091	29004226	*LOC105372763, LINC00113*	A	1.69	1.3×10^{-5}	1.69	7.1×10^{-4}	A	1.24	0.041
21	rs9976476	29017708	*LOC105372763*	G	1.65	1.7×10^{-5}	1.71	3.2×10^{-4}	G	1.17	0.131
21	rs148082602†	40685515	*BRWD1*	G	1.48	0.0016	1.88	4.3×10^{-5}	-	-	-
21	rs78367934†	40786828	*LCA5L*	G	1.76	9.7×10^{-5}	2.02	9.7×10^{-5}	-	-	-
21	rs2837017	40788083	*LCA5L*	A	1.75	1.2×10^{-4}	1.99	1.2×10^{-4}	A	1.08	0.514
21	rs73906034	40788127	*LCA5L*	A	1.79	3.2×10^{-4}	2.31	1.9×10^{-5}	A	1.24	0.119
21	rs540798	40960096	*B3GALT5*	A	1.46	0.0019	1.86	5.7×10^{-5}	A	1.00	0.967
21	rs117674297╥	42343366	*DSCAM, BACE2*	A	2.96	2.6×10^{-6}	2.05	0.0190	-	-	-
22	rs5994705	33647908	*LARGE*	C	1.66	2.6×10^{-5}	1.56	0.0034	C	0.99	0.915

Abbreviations: Chr, chromosome; SNP, single nucleotide polymorphism; OR, odds ratio.

ӜLocation refers to human genome build 37 (GRCh37/hg19) coordinates.

**Corresponding locus shown for SNPs within genes; two nearest genes shown for intergenic SNPs.

‡ORs for A1 allele in logistic regression under additive model, with three principal components and gender as covariates.

₸ORs for A1 allele under additive model in logistic regression, with sex as a covariate.

†SNP did not settle in the iPLEX replication genotyping platform.

╥SNP was monomorphic or had a very low minor allele frequency (<0.01) in replication population.

±SNP deviated from Hardy–Weinberg equilibrium ($p < 0.001$) in replication analysis.

*SNP was not genotyped in replicate analysis for technical reasons.

Supplementary Table S2. Areas (20 kb) around SNPs that have been suggested to play a role in bronchiolitis susceptibility in previous studies screened in our GWAS data. SNPs with $p \leq 0.01$ (either in GWAS or RSV GWAS) are shown. The exact SNPs previously reported to be associated with bronchiolitis did not show associations or were not present in our data. The best variant was rs56039226 in *CX3CR1* intron ($p = 5.7 \times 10^{-5}$).

SNP information					MAF in GWAS			GWAS		RSV GWAS	
Chr	SNP	LocationӜ	Genes	A1	cases	RSV cases	controls	OR‡	*p*	OR‡	*p*
1	rs2494251	159281151	3 kb 3' of *FCER1A*	A	0.408	0.409	0.469	0.73	0.0091	0.72	0.0377
2	2:191844678:TC:T	191844678	*STAT1* intron	T	0.026	0.029	0.008	3.89	0.0016	5.22	8.4×10^{-4}
3	rs56039226	39318192	*CX3CR1* intron	A	0.041	0.043	0.011	4.43	5.7×10^{-5}	4.17	9.8×10^{-4}
4	rs201853366	38768759	5 kb 3' of *TLR10*	T	0.060	0.069	0.040	1.91	0.0125	2.31	0.0068
4	rs12503707	74590689	15,5 kb 5' of *IL8*	T	0.028	0.029	0.011	2.95	0.0071	3.41	0.0111
6	rs9267492	31521481	*NFKBIL1* intron; 7 kb 5' of *LTA*	T	0.036	0.042	0.014	2.66	0.0072	2.90	0.0107
6	rs2230365	31525448	*NFKBIL1*; 3 kb 5' of *LTA*	T	0.171	0.176	0.133	1.57	0.0060	1.67	0.0130
6	rs34877047	31525738	*NFKBIL1* intron; 3 kb 5' of *LTA*	T	0.029	0.031	0.014	2.37	0.0226	3.32	0.0087
6	rs4947326	31533718	6 kb 5' of *LTA*	G	0.034	0.038	0.012	2.85	0.0059	2.95	0.0140
6	rs4947327	31533728	6 kb 5' of *LTA*	A	0.034	0.038	0.012	2.85	0.0059	2.95	0.0140
6	rs9267497	31534500	5 kb 5' of *LTA*	G	0.034	0.038	0.012	2.85	0.0059	2.95	0.0140
6	rs1799964	31542308	200 bp 3' of *LTA*	C	0.205	0.226	0.169	1.41	0.0161	1.58	0.0094
6	rs1052248	31556581	*LST1* 3'UTR; near *NRC3;* 13.5 kb of *TNF*	A	0.269	0.302	0.227	1.40	0.0108	1.63	0.0034
6	rs3087617	31556656	*LST1* 3'UTR; near *NRC3;* 11 kb 3' of *TNF*	T	0.090	0.095	0.056	1.85	0.0050	1.82	0.0271
6	rs34744948	31562767	17 kb 3' of *TNF;* 2 kb 5' of *NRC3*	C	0.108	0.105	0.072	1.73	0.0054	1.62	0.0538
6	rs71563353	31562924	17 kb 3' of *TNF;* 2 kb 5' of *NRC3*	T	0.108	0.105	0.072	1.73	0.0054	1.62	0.0538
6	6:52064298	52064298	9 kb 3' of *IL17A*	C	0.026	0.038	0.011	2.78	0.0093	4.44	5.3×10^{-4}
8	rs118073036	22027130	*BMP1* intron, 5 kb 3' of *SFTPC*	A	0.059	0.049	0.028	2.88	1.5×10^{-4}	2.37	0.0191
9	rs79896024	120492632	13kb 3' of *TLR4*	G	0.035	0.046	0.016	2.29	0.0129	2.79	0.0053
10	rs10887238	81716268	*SFTPD* intron	A	0.057	0.059	0.026	2.24	0.0027	2.34	0.0106
16	rs147427210	27318922	6.1 kb 5' of *IL4R*	G	0.031	0.014	0.011	3.49	0.0041	1.64	0.4591
16	rs145115415	27388083	12 kb 3' of *IL4R*	T	0.028	-	0.006	1.66	0.0023	-	-
17	rs28943568	26082645	1.1 kb 3' of *NOS2*	A	0.066	0.088	0.039	2.05	0.0044	2.83	2.9×10^{-4}
17	rs11653716	26084532	*NOS2* intron	G	0.101	0.046	0.069	2.29	0.0129	2.88	0.0053
17	rs200393040	34204173	*CCL5* intron	CT	0.031	0.047	0.016	2.03	0.047	2.76	0.0076
20	rs17711842	3636929	3 kb 3' of *GFRA4;* 12 kb 3' of *ADAM33*	T	0.069	0.089	0.035	1.81	0.0157	2.25	0.0040
20	rs7266815	3639077	0.9 kb 3' of *GFRA4;* 10 kb 3' of *ADAM33*	T	0.069	0.090	0.035	1.83	0.0141	2.29	0.0035
20	rs7266457	3644478	0.5 kb 5' of *GFRA4;* 4 kb 3' of *ADAM33*	T	0.076	0.098	0.036	2.01	0.0036	2.53	7.8×10^{-4}
20	rs369784662	3645117	1 kb 5' of *GFRA4;* 3.5 kb 3' of *ADAM33*	A	0.075	0.098	0.036	1.98	0.0047	2.49	9.5×10^{-4}
20	rs41530048	3651497	*ADAM33* intron	A	0.079	0.103	0.035	2.08	0.0020	2.60	4.2×10^{-4}
20	rs3918393	3653642	*ADAM33* intron	T	0.072	0.095	0.035	1.85	0.0109	2.32	0.0023
20	rs150996533	3664915	2 kb 5' of *ADAM33*	G	-	0.103	0.044	-	-	2.23	0.0033

20	rs78761371	3665371	3 kb 5' of *ADAM33*; 2,2 kb 3' of *SIGLEC1*	G	-	0.103	0.043	-	-	2.28	0.0026
20	rs17701662	3670512	*SIGLEC1* intron; 8 kb 5' of *ADAM33*	T	-	0.102	0.044	-	-	2.22	0.0033
21	rs143991016	42809489	*MX1* intron	G	0.030	0.029	0.012	3.05	0.0038	2.96	0.0233
21	rs113340440	42811078	*MX1* intron	A	0.049	0.058	0.021	2.61	0.0016	2.94	0.0023
21	rs77214232	42812445	*MX1* intron	T	0.032	0.033	0.012	2.94	0.0039	2.87	0.0212
21	rs2238714	42815008	*MX1* intron	C	0.035	0.033	0.012	3.06	0.0024	2.87	0.0212
21	rs2075804	42815974	*MX1* intron	A	0.035	0.033	0.012	3.06	0.0024	2.87	0.0212
21	rs34937035	42817245	*MX1* intron	C	0.035	0.033	0.012	3.35	0.0012	3.24	0.0108
21	rs192084957	42818272	*MX1* intron	C	0.035	0.033	0.012	3.06	0.0024	2.87	0.0212
21	rs74655087	42818844	*MX1* intron	A	0.035	0.033	0.012	3.06	0.0024	2.87	0.0212
21	rs74429776	42819339	*MX1* intron	A	0.035	0.033	0.012	3.06	0.0024	2.86	0.0215
21	rs116868083	42834988	1.5 kb 3' of *TMPRSS2;* 4 kb 3' of *MX1*	A	0.021	-	0.007	3.94	0.0038	-	-
21	rs8133614	42835739	5 kb 3’ of *MX1;* 1 kb 3’ of *TMPRSS2*	G	0.023	0.025	0.010	3.37	0.0076	3.89	0.0086
21	rs12627374	42837691	5 kb 3’ of *MX1;* 3’ UTR of *TMPRSS2*	T	0.021	-	0.008	3.34	0.0098	-	-
21	rs80325088	46329175	*ITGB2* intron	T	0.046	0.043	0.024	2.29	0.0076	2.09	0.0646

Abbreviations: Chr, chromosome; SNP, single nucleotide polymorphism; OR, odds ratio; MAF, minor allele frequency.

ӜLocation refers to human genome build 37 (GRCh37/hg19) coordinates.

‡ORs for A1 allele in logistic regression under additive model, with three principal components and gender as covariates.

Supplementary Table S3. Areas (20 kb) around SNPs that have been suggested to play a role in asthma susceptibility in previous studies (SNPs in the NHGRI-EBI GWAS Catalog with $p \leq 10^{-6}$) screened in our GWAS data. SNPs with $p \leq 0.0001$ either in GWAS, RSV GWAS, or GWAS <1 year are shown.

Variant details					GWAS		RSV GWAS		GWAS <1 year	
SNP	Chr	LocationӜ	Genes	A1	OR‡	*p*	OR‡	*p*	OR‡	*p*
rs13182546	5	82831359	*VCAN* intron	G	1.813	$2.4×10^{-5}$	-	-	-	-
rs13159438	5	82839305	*VCAN* intron	T	1.817	$2.2×10^{-5}$	1.906	$2.6×10^{-4}$	1.779	$2.2×10^{-3}$
rs529998	5	82841764	*VCAN* intron	T	1.473	0.0010	1.952	$1.7×10^{-5}$	-	-
rs647873	5	82843440	*VCAN* intron	C	-	-	1.925	$2.0×10^{-5}$	-	-
rs9271499	6	32589291	6.7kb 5' of *HLA-DQA1*	T	1.517	$3.7×10^{-4}$	1.670	$6.1×10^{-4}$	1.766	$2.0×10^{-5}$
rs9271500	6	32589300	6.7kb 5' of *HLA-DQA1*	T	1.452	0.0012	1.555	0.0028	1.692	$6.2×10^{-5}$
rs9271501	6	32589301	6.7kb 5' of *HLA-DQA1*	G	1.452	0.0012	1.555	0.0028	1.692	$6.2×10^{-5}$
rs9271502	6	32589305	6.7kb 5' of *HLA-DQA1*	A	1.523	$3.3×10^{-4}$	1.676	$5.6×10^{-4}$	1.772	$1.8×10^{-5}$
rs9271566	6	32590443	5.5kb 5' of *HLA-DQA1*	G	1.573	$7.8×10^{-5}$	1.697	$3.1×10^{-4}$	1.821	$5.2×10^{-6}$
rs9271567	6	32590456	5.5kb 5' of *HLA-DQA1*	T	1.573	$7.8×10^{-5}$	1.697	$3.1×10^{-4}$	1.821	$5.2×10^{-6}$
rs9271597	6	32591291	4.7kb 5' of *HLA-DQA1*	A	1.459	$6.9×10^{-4}$	1.585	0.0011	1.650	$7.5×10^{-5}$
rs9271600	6	32591332	4.6kb 5' of *HLA-DQA1*	G	1.478	$4.7×10^{-4}$	1.601	$9.0×10^{-4}$	1.665	$5.8×10^{-5}$
rs9271601	6	32591337	4.6kb 5' of *HLA-DQA1*	T	1.478	$4.7×10^{-4}$	1.601	$9.0×10^{-4}$	1.665	$5.8×10^{-5}$
rs142530081	6	32606682	*HLA-DQA1* intron	G	2.561	$3.1×10^{-5}$	1.581	0.1419	2.030	0.0079
rs36223157	6	32606691	*HLA-DQA1* intron	G	2.561	$3.1×10^{-5}$	1.581	0.1419	2.030	0.0079
rs1130153	6	32610947	*HLA-DQA1* 3'-UTR	C	2.716	$7.7×10^{-6}$	1.732	0.0725	2.260	0.0018
rs9282052	6	32613360	*HLA-DQA1* 3'-UTR	CA	1.790	$1.4×10^{-4}$	-	-	1.996	$5.6×10^{-5}$
rs28633411	6	32613362	*HLA-DQA1* intron	A	2.580	$2.9×10^{-5}$	1.634	0.1164	2.076	0.0063
rs28375404	6	32614917	*HLA-DQA1* intron	T	2.557	$2.8×10^{-5}$	1.671	0.0930	2.067	0.0058
rs28819191	6	32622110	*HLA-DQA1* intron	A	2.659	$1.4×10^{-5}$	1.619	0.1231	2.157	0.0036
6:32631070:T:C	6	32631070	*HLA-DQB1* intron	C	2.690	$7.4×10^{-5}$	2.464	0.0030	2.357	0.0023
rs73729452	6	32632547	*HLA-DQB1* intron	C	2.448	$7.0×10^{-5}$	1.434	0.2581	1.970	0.0105
rs9788617	14	51244776	*NIN* intron	G	1.277	0.0339	1.814	$5.7×10^{-5}$	1.367	0.0160
rs7143959	14	51245666	*NIN* intron	T	1.317	0.0158	1.865	$2.0×10^{-5}$	1.413	0.0071
rs34148215	14	51249238	*NIN* intron	T	1.290	0.0270	1.804	$6.1×10^{-5}$	1.376	0.0136
rs764964	14	51254908	*NIN* intron	T	1.322	0.0198	1.887	$3.2×10^{-5}$	1.440	0.0065
rs11157766	14	51262497	*NIN* intron	A	1.374	0.0073	1.950	$1.3×10^{-5}$	1.461	0.0045
rs12881609	14	51263301	*NIN* intron	C	1.399	0.0048	1.980	$8.3×10^{-6}$	1.485	0.0033

SNP, single nucleotide polymorphism; GWAS, genome-wide association study; Chr, chromosome; OR, odds ratio.

ӜLocation refers to human genome build 37 (GRCh37/hg19) coordinates.

‡ORs for A1 allele in logistic regression under additive model, with three principal components and gender as covariates

Supplementary Table S4. Meta-analyses[§] for SNPs that had a *p* value of <0.1 in the Dutch replicate and ORs in the same direction with GWASs.

Variant details					GWAS		RSV GWAS		GWAS < 1 year		Replication, NL		Meta-analysis: NL, GWAS		Meta-analysis: NL, RSV GWAS		Meta-analysis: NL, GWAS <1year	
SNP	Chr	LocationӜ	Genes**	A1	OR‡	*p*	OR‡	*p*	OR‡	*p*	OR₸	*p*	OR	*p*	OR	*p*	OR	*p*
rs269101	1	112628860	*LOC643355, KCND3*	T	1.605	4.2×10^{-5}	1.703	3.0×10^{-4}	1.599	2.4×10^{-4}	1.213	0.0541	1.368	3.5×10^{-5}	1.350	2.9×10^{-4}	1.348	1.5×10^{-4}
rs269094	1	112639174	*LOC643355, KCND3*	C	1.672	5.3×10^{-5}	1.809	1.9×10^{-4}	1.757	5.2×10^{-5}	1.281	0.0292	1.442	1.5×10^{-5}	1.439	8.1×10^{-5}	1.453	2.2×10^{-5}
rs10738377	9	14757744	*FREM1*	A	0.619	8.9×10^{-4}	0.487	3.3×10^{-4}	0.662	0.0085	0.816	0.0657	0.737	5.0×10^{-4}	0.723	8.2×10^{-4}	0.761	0.0025
rs10756611	9	14765611	*FREM1*	G	0.534	2.6×10^{-5}	0.431	4.1×10^{-5}	0.574	6.1×10^{-4}	0.813	0.0638	0.699	6.2×10^{-5}	0.704	3.3×10^{-4}	0.727	5.2×10^{-4}
rs9591920	13	59315119	*LOC105370220*	T	2.162	2.0×10^{-6}	2.033	3.5×10^{-4}	1.853	7.1×10^{-4}	1.405	0.0227	1.712	9.8×10^{-7}	1.606	7.1×10^{-5}	1.570	9.2×10^{-5}
rs1537091	21	29004226	*LOC105372763, LINC00113*	A	1.691	1.3×10^{-5}	1.693	7.1×10^{-4}	1.606	4.5×10^{-4}	1.240	0.0408	1.418	1.1×10^{-5}	1.367	3.3×10^{-4}	1.367	1.6×10^{-4}

Abbreviations: SNP, single nucleotide polymorphism; Chr, chromosome; OR, odds ratio; NL, Dutch replication population.

§Meta-analyses performed under fixed-effects model for logistic regression result files of discovery and replication data.

ӜLocation refers to human genome build 37 (GRCh37/hg19) coordinates.

**Corresponding locus shown for SNPs within genes; two nearest genes shown for intergenic SNPs.

‡ORs for A1 allele in logistic regression under additive model, with three principal components and gender as covariates.

₸ORs for A1 allele under additive model in logistic regression, with sex as a covariate.

Supplementary Table S5. SNPs that had a p-value < 0.05 in the Dutch replication population (NL) were further studied in the Finnish case set from Turku, with NordicDB population controls as a reference.

Variant information					GWAS		Replication, NL		Replication, Turku (FIN)	
SNP	Chr	LocationӜ	Genes**	A1	OR‡	*p*	OR₸	*p*	OR₸	*p*
rs269094	1	112639174	*LOC643355, KCND3*	C	1.672	5.3×10^{-5}	1.281	0.0292	0.989	0.9352
rs9591920	13	59315119	*LOC105370220*	T	2.162	2.0×10^{-6}	1.405	0.0227	1.469	0.0415
rs1537091	21	29004226	*LOC105372763, LINC00113*	A	1.691	1.3×10^{-5}	1.240	0.0408	1.167	0.2290

Abbreviations: GWAS, genome-wide association study; NL, Dutch population; SNP, single nucleotide polymorphism; Chr, chromosome; OR, odds ratio.

ӜLocation refers to human genome build 37 (GRCh37/hg19) coordinates.

**Corresponding locus shown for SNPs within genes; two nearest genes shown for intergenic SNPs.

‡ORs for A1 allele in logistic regression under additive model, with three principal components and gender as covariates.

₸ORs are for A1 allele under additive model in logistic regression with sex as a covariate.

Supplementary Table S6. Arrays used to genotype GWAS discovery populations.

GWAS population	Genome-wide genotyping method
Kuopio (FIN), Gothenburg (SWE)	HumanOmniExpress BeadChip, Illumina
Tampere cases (FIN)	Infinium HumanCoreExome BeadChip, Illumina
NordicDB population controls (FIN)	HumanCNV-370-v1.0, Illumina

Abbreviations: GWAS, genome-wide association study; FIN, Finland; SWE, Sweden
